# The *N*-methyl-d-aspartate receptor hypothesis of ketamine’s antidepressant action: evidence and controversies

**DOI:** 10.1098/rstb.2023.0225

**Published:** 2024-07-29

**Authors:** Yihao Jiang, Yiyan Dong, Hailan Hu

**Affiliations:** ^1^ Department of Affiliated Mental Health Center & Hangzhou Seventh People’s Hospital and School of Brain Science and Brain Medicine, Zhejiang University School of Medicine, Hangzhou 310058, People's Republic of China; ^2^ Nanhu Brain-Computer Interface Institute, MOE Frontier Science Center for Brain Science and Brain-Machine Integration, State Key Laboratory of Brain-Machine Intelligence, New Cornerstone Science Laboratory, Zhejiang University, Hangzhou 311100, People's Republic of China

**Keywords:** ketamine, depression, NMDAR hypothesis, NMDAR inhibitors, antidepressant efficacy

## Abstract

Substantial clinical evidence has unravelled the superior antidepressant efficacy of ketamine: in comparison to traditional antidepressants targeting the monoamine systems, ketamine, as an *N*-methyl-d-aspartate receptor (NMDAR) antagonist, acts much faster and more potently. Surrounding the antidepressant mechanisms of ketamine, there is ample evidence supporting an NMDAR-antagonism-based hypothesis. However, alternative arguments also exist, mostly derived from the controversial clinical results of other NMDAR inhibitors. In this article, we first summarize the historical development of the NMDAR-centred hypothesis of rapid antidepressants. We then classify different NMDAR inhibitors based on their mechanisms of inhibition and evaluate preclinical as well as clinical evidence of their antidepressant effects. Finally, we critically analyse controversies and arguments surrounding ketamine’s NMDAR-dependent and NMDAR-independent antidepressant action. A better understanding of ketamine’s molecular targets and antidepressant mechanisms should shed light on the future development of better treatment for depression.

This article is part of a discussion meeting issue ‘Long-term potentiation: 50 years on’.

## 1. Introduction

Classical antidepressants, including selective serotonin reuptake inhibitors (SSRI), were developed based on the monoamine deficiency hypothesis of depression [1]. However, their delayed onset and limited efficacy [2,3] suggest that classical antidepressants may not target directly at the core pathophysiology mechanism of depression. Ketamine, by contrast, has emerged in recent decades as a rapid-acting and highly potent antidepressant [4–6]. Intravenous ketamine therapy for depression has been argued to be the most vital breakthrough in the psychiatry field since the 1950s [7]. Hence, what ketamine targets, and how ketamine works, for its antidepressant activity, is at the heart of understanding the mechanism of depression, and developing new-generation antidepressant treatments.

Ketamine is primarily an antagonist of the glutamate receptor *N*-methyl-d-aspartate receptor (NMDAR) [8,9]. Historically, even before the discovery of ketamine, NMDARs have been implicated several times to be a target for rapid antidepressants [10]. Thus, it was naturally proposed that ketamine may act by blocking NMDARs to execute its antidepressant actions [4,11,12]. This NMDAR hypothesis has garnered plenty of favourable experimental support [13–19]. However, some doubts have also been cast on the NMDAR hypothesis, as many NMDAR inhibitors have failed to show antidepressant effects in clinical trials [20] (but see recent developments of AXS-05 (brand name Auvelity) [21–23]). Moreover, data from some ketamine metabolites and enantiomers in preclinical studies also raised an alternative hypothesis of ketamine’s targets [24,25]. The goal of this article is to delve into the evidence and arguments surrounding the NMDAR hypothesis of ketamine, hoping to clarify the debates on how ketamine works to combat depression.

## 2. Initial discoveries of the *N*-methyl-d-aspartate receptors as a target for rapid antidepressant actions

The initial implication of NMDARs as a potential target responsible for rapid antidepressant effects dates back to an unexpected clinical case report in 1961. In this study, Dr George Crane accidentally discovered that, in tuberculosis patients suffering from depressive disorders, d-cycloserine (DCS), a tuberculosis antibiotic, rapidly improved mood [26]. He commented that ‘It is difficult to explain why psychiatric benefits should have occurred almost immediately following drug administration’ [26], although later trials have yielded mixed results [27,28]. Further investigations revealed that DCS is a partial agonist at the NMDAR glycine site, and at the dosage used for tuberculosis treatment, 500 mg d^−1^ [26], DCS may partially antagonize NMDAR function [20,29].

The second set of evidence for NMDAR-dependent antidepressant mechanisms, generated in animal models, came in the 1990s. Based on the observations that inescapable shocks induced depression-like behaviours are accompanied by altered NMDAR-dependent plasticity in the hippocampus [10], Drs Skolnick and Trullas speculated that NMDAR antagonists may represent a novel class of antidepressants [30]. A series of follow-up experiments were performed, using either an NMDAR open channel blocker—dizocilpine (MK-801), a competitive NMDAR antagonist—2-amino-7-phosphonoheptanoic acid (AP-7), or an NMDAR glycine-site partial agonist—1-aminocyclopropanecarboxylic acid (ACPC), which all function as NMDAR inhibitors. In the forced swim test (FST), an assay to measure depressive-like behaviours, these drugs all rapidly alleviate depression-like phenotypes within 15 min [30]. Such effects in animal models were further confirmed by other groups [31,32]. In addition, although classical antidepressants are clinically deficient in rapid action, 16 out of 17 of them, when chronically treated, can biochemically reduce the agonist binding affinity of NMDARs [33]. This attenuated ligand binding affinity of NMDARs is specific to antidepressants and not induced by non-antidepressant psychotropic drugs [33]. Such data indicate that NMDAR antagonism may be a common pathway for antidepressant actions.

Despite the above evidence from preclinical work, the first clinical evidence supporting the NMDAR theory of rapid antidepressants did not arrive until 2000 when Dr John Krystal and Dennis Charney’s team serendipitously uncovered in major depressive disorder (MDD) patients after a single intravenous (i.v.) infusion of subanesthetic (0.5 mg kg^−1^) ketamine a strikingly rapid (within 4 h) and durable (at least 3 days for Hamilton Depression Rating Scale (HDRS), and 1–2 weeks for depressed mood) antidepressant effects [4,6,34].

Since then, ketamine—initially discovered as an anaesthetic and later on the well-known hallucinogenic ‘party drug’—has opened a new era of rapid anti-depression research [4,6]. Soon afterwards, Dr Carlos Zarate’s team reported the detailed efficacy of a single dose of ketamine in treatment-resistant depression (TRD) patients [5]. They identified a more accurate antidepressant onset of ketamine—within 110 min for HDRS, and 40 min for depressed mood and guilt [5]. They also identified a surprisingly sustained responsive rate: compared to 0% in the placebo group, ketamine’s effects last for at least 1 week for 35% of patients and at least 2 weeks for 11% of patients [5]. These rapid and sustained antidepressant effects of ketamine were widely replicated by a subsequent series of reports [20,35–38]. Importantly, ketamine also reduced suicidal ideation in a rapid (within 40 min) and sustained (up to 1 week) manner [39,40]. Further studies also expanded the clinical application of ketamine to the depressive episodes of treatment-resistant bipolar disorders [41], psychotic depression [42–44] and negative symptoms in schizophrenia (e.g. anhedonia and suicidal ideation) [45,46].

## 3. Preclinical and clinical evidence of antidepressant activity of other *N*-methyl-d-aspartate receptor inhibitors

**Figure 1.**
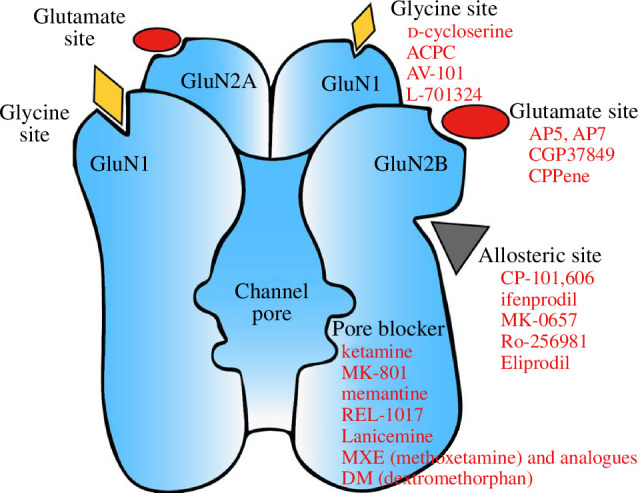
Drug binding sites of NMDAR and related inhibitors. NMDAR mainly has four types of binding sites: glutamate site, glycine site, GluN2B-selective allosteric site, and open channel blocking sites. Drugs recognizing these different sites are depicted under each category.

In addition to the aforementioned drugs, multiple other NMDAR inhibitors were also tested for antidepressant activity in preclinical (table 1) or clinical (table 2) studies. Before reviewing their effects in detail, we briefly summarize the inhibition mechanisms of different classes of NMDAR inhibitors and highlight the unique properties of ketamine. As an ion channel, the NMDARs are heterotetramers composed of two GluN1 and two GluN2 (typically NR2A or NR2B) subunits [101]. Based on the recognition site, NMDAR inhibitors can be broadly classified into at least four groups: those acting on (i) the glutamate site on GluN2 subunits, (ii) the glycine site on GluN1, (iii) the allosteric sites on GluN2B, and (iv) the phencyclidine (PCP) site or the magnesium (Mg^2+^) site located within the channel pore (figure 1; table 3; [120–123]).

**Table 1.** Antidepressant efficacy of NMDAR inhibitors in preclinical studies. (FST, forced swim test; FUST, female urine sniff test; LH, learned helpnessess test; NA, not applicable; SIT, social interaction test; SPT, sucrose preference test; TST, tail suspension test.)

inhibitor type	drug	animal model	behavioural assay	antidepressant efficacy		references
				rapid	sustained	
open channel blocker	ketamine	naive mice, rats	FST, TST	30 min–3 h	1 day–2 weeks	[13–15,19,47–55]
			SPT	30 min	NA	[15,49]
			FUST	NA	1 day	[56]
		depressed mice, rats	FST, TST	30 min–6 h	12 h–1 day	[15,17,57]
			SPT	30 min–1 h	1 day–1 week	[15,17,57,58]
			LH	30 min–1 h	1 day–1 week	[13–15,17,47,55]
			SIT	NA	1 day	[55]
			FUST	NA	2 days	[56]
	(S)-ketamine	naive mice	FST	1 h	1 day	[55]
		depressed mice	LH	NA	1 day	[55]
			FST, TST	NA	1–7 days	[59,60]
			SPT	NA	1–7 days	[59,60]
	(R)-ketamine	naive mice	FST	1 h	1 day	[55]
		depressed mice, rats	FST, TST	NA	1–7 days	[59,60]
			LH	NA	1–5 days	[55,60]
			SPT	NA	1–7 days	[59,60]
	methoxetamine (MXE)	naive mice	FST, TST	30 min	1 day	[49]
			SPT	30 min	NA	[49]
	MXE analogues	naive mice	FST, TST	30 min	NA	[50]
	MK-801	naive mice, rats	FST, TST	15 min–3 h	no effect	[13,15,30,51–53,55,61]
		depressed mice	SIT	NA	no effect	[55]
			FST, TST	6 h	1 day	[62,63]
			SPT	NA	2–4 days	[62,63]
	lanicemine (AZD6765)	naive mice	FST	45 min	NA	[53]
	memantine	naive mice, rats	FST, TST	30 min–4 h	NA	[64–66]
			FST	no effect	no effect	[67]
		depressed rats	FST	1 h	NA	[68]
	dextromethorphan (DM)	naive mice	FST, TST	30 min	NA	[54,69–71]
	D-methadone	naive rats	FUST	NA	1 day	[56]
		depressed rats	FUST	NA	2 days	[56]
glutamate site competitive antagonist	AP7	naive mice	FST	30 min	NA	[30]
	CGP37849	naive rats	FST	10 min–1 h	NA	[32]
	CPPene (midafotel)	naive mice	FST	30 min	1 day	[15]
glycine site competitive antagonist	ACPC	naive mice, rats	FST, TST	10 min–1 h	NA	[30,32,53]
	AV-101	naive mice	FST, TST	1 h	1 day	[47]
		depressed mice	LH	NA	1 day–1 week	[47]
	L-701324	naive mice	FST, TST	30 min	NA	[72]
		depressed mice	FST, TST	NA	1–2 days	[72]
			SPT	NA	1 week	[72]
GluN2B-selective allosteric antagonist	eliprodil	naive mice	FST	1 h	NA	[31]
	Ro-256981	naive mice, rats	FST, TST	20 min–1 h	1 day	[13,14,19,52,53,61]
		depressed rats	SPT	NA	1 day–1 week	[58]
	CP-101,606	naive mice	FST	1 h	NA	[73]
	ifenprodil	depressed rats	FST	NA	2 days	[74]
			SPT	30 min	NA	[74]

**Table 2.** Antidepressant efficacy of NMDAR inhibitors in clinical studies. (BDD, bipolar depressive disorder; i.n., intranasal; i.v., intravenous; NA, not applicable; p.o., oral. Notes: all other studies are randomized, double-blind, placebo-controlled; +, have a significant antidepressant efficacy; −, without a statistical significant difference to control.)

type	drug	patient	dosing regimen	antidepressant efficacy		effic acy	references
				onset time	peak response		
open channel blocker	ketamine	MDD (*n* = 7), TRD (*n*= 18/73), BDD (*n* =15)	i.v., single dose, 0.5 mg kg^−1^	40 min	45.8–71% versus 0–28%	+	[4,5,20,35–38]
	(S)-ketamine	TRD (*n* = 30)	i.v., single dose, 0.2 mg kg^−1^ or 0.4 mg kg^−1^	2 h	67% versus 0%	+	[75]
		TRD (*n* = 67/68)	i.n., twice weekly, 84 mg	2–4 h	50% versus 10%	+	[76,77]
		TRD (*n* = 138)	i.n., twice weekly, 28–84 mg	NA	27% versus 13%	−	[78]
		TRD (*n* = 346)	i.n., twice weekly, 56 or 84 mg	24 h	27% versus 13%	−	[79]
		TRD (*n* = 197)	i.n., twice weekly, 56–84 mg	24 h	69% versus 52%	+	[80]
	(R)-ketamine	TRD (*n* = 7)	i.v., single dose, 0.5 mg kg^−1^	1 h	100%	+	[81]
		TRD (*n* = 10)	i.v., single dose, 0.5 mg kg^−1^	NA	20% versus 10%	−	[82]
	memantine	MDD (*n* = 32)	p.o., initially 5 mg d^−1^, titrated for 3 weeks to 20 mg d^−1^	NA	13% versus 13%	−	[5]
		MDD^b^ (*n* = 80)	p.o., initially 5 mg d^−1^, titrated for 3 weeks to 20 mg d^−1^	1 month	76% versus 72% escitalopram	+	[83]
		MDD (*n* = 31)	p.o., initially 5 mg d^−1^, titrated for 3 weeks to 20 mg d^−1^	NA	13% versus 19%	−	[84]
		BDD (*n* = 29)	p.o., initially 5 mg d^−1^, titrated for 3 weeks to 20 mg d^−1^	4 weeks	57% versus 20%	−	[85]
		MDD (*n* = 8)	p.o., initially 5 mg d^−1^, titrated for 3 weeks to 20–40 mg d^−1^	1 week	63%	+	[86]
		MDD (*n* = 62)	p.o., initially 10 mg d^−1^, titrated for 1 week to 20 mg d^−1^	2 weeks	90% versus 48%	+	[87]
	dextromethorphan (DM)	TRD (*n* = 20)	p.o., initially 20 mg d^−1^, titrated for 2 weeks to 90 mg d ^−1^	4 weeks	64%	+	[88]
		BDD (*n* = 309)	p.o., open-label VPA plus placebo, 30 mg d^−1^ DM, 60 mg d^−1^ DM	12 weeks	NA	+	[89]
		MDD^b^ (*n* = 80)	p.o., AXS-05 (45 mg DM + 105 mg bupropion) versus bupropion 105 mg	1 week	16% versus 3%	+	[22]
		MDD (*n*=327)	p.o., AXS-05 (45 mg DM + 105 mg bupropion) versus placebo	1 week	39.5% versus 17.30%	+	[21]
	DM+memantine	moderate mood symptoms (*n*=312)	four groups: DM (30 mg d^−1^) + memantine (5 mg d^−1^), DM (30 mg/day), memantine (5 mg d^−1^), or placebo. Valproate as an add-on.	12 weeks	59.1% versus 56.50%	+	[90]
	lanicemine (AZD6765)	TRD (*n* = 22)	i.v., single dose, 150 mg	80 min	32% versus 15%	+	[91]
		TRD (*n* = 152)	i.v., single dose, 100 mg	1 h	NA	+	[92]
		TRD (*n* = 152)	i.v., 100 mg, 3 infusions per week	2 weeks	37% versus 16%	+	[92]
		TRD (*n* = 302)	i.v., 100 mg, 15 infusions over 12 weeks	NA	44% versus 39%	−	[93]
	REL-1017 (esmethadone)	TRD (*n* = 62)	p.o., REL-1017 (25 mg or 50 mg orally once a day)	4 days	39% versus 5%	+	[94]
NR2B selective allosteric inhibitor	CP-101,606 (Traxoprodil)	TRD (*n* = 30)	i.v., single dose, 0.75 mg kg^−1^ with paroxetine as add on	4 days	60% versus 20%	+	[95]
	MK-0657 (CERC-301)	TRD (*n* = 5)	p.o., MK-0657 monotherapy (4–8 mg d^−1^) or placebo	5 days	20% versus 0%	+	[96]
glycine site competitive antagonist	d-cycloserine (DCS)	TRD (*n* = 22)	250 mg d^−1^ d-cycloserine added to their ongoing antidepressant medications	2 weeks	54% versus 15%	−	[28]
		TRD (*n* = 26)	p.o., initially 250 mg d^−1^, and titrated for 4 weeks to 1000 mg d^−1^	6 weeks	54% versus 15%	+	[27]
	NRX-101 (DCS with lurasidone)	BDD (*n* = 22)	p.o., NRX-101 combined with ketamine infusion	14 days	100% versus 60%	+	[97]
	AV-101 (L-4-chlorokynurenine)	TRD (*n* = 19)	p.o., AV-101 monotherapy 1080 mg d^−1^ for 7 days, then 1440 mg d^−1^ for the next 7 days	No	0% versus 5.3%	−	[98]
unknown site antagonist	nitrous oxide (N_2_O)	TRD (*n* = 20)	1 h inhalation of 50% nitrous oxide/50% oxygen or 50% nitrogen/50% oxygen (placebo control)	2 h	20% versus 5%	+	[99]
		TRD (*n* = 24)	a single 1 h inhalation with (i) 50% nitrous oxide, (ii) 25% nitrous oxide, or (iii) placebo (air/oxygen)	2 h	42% versus 11%	+	[100]

^a^ Open-label, non-controlled study.

^b^ Randomized, double-blind, active-controlled study.

**Table 3.** Ketamine’s affinity to varied receptors. (Notes*:* Ki is the dissociation constant; IC50 or EC50 corresponds to the concentration of ketamine required to reduce or potentiate the receptor’s activity to half of the saturated concentration value.)

receptor	effect (μM)	references
NMDAR	Ki = 0.18–4.9; IC50 = 0.43– 8.2	[102–105]
5-HT2 receptor	Ki = 15	[106]
serotonin transporter	IC50 = 18.8	[107]
hyperpolarization-activated cyclic nucleotide-gated channel (HCN) channel	IC50 = 8.2–15.6	[108]
μ opioid receptor	Ki = 4.38–42.1; EC50 = 9– 34	[109–112]
κ opioid receptor	Ki = 25–28.1; EC50 = 16–29	
δ opioid receptor	Ki = 272	
M1 mAChR	IC50 = 5.7	[105,113,114]
α3β4 nAChR	IC50 = 3–9.5	
other nAChR	IC50 = 17–92	
oestrogen receptors (ERs)	EC50 = 2.3	[115]
dopamine transporters (DATs)	IC50 = 4.6	[116]
dopamine D2 receptors (D2Rs)	Ki = 0.05–1; EC50 = 0.4–2	[106,117,118]
tyrosine kinase receptor 2 (TRKB)	Ki = 2.86–12.30	[119]

Ketamine, together with MK-801, PCP and memantine, belongs to the fourth group—the channel blockers acting on the PCP/Mg^2+^ site [122]. These NMDAR channel blockers, also known as uncompetitive open channel trapping blockers, act in a use-dependent manner, blocking the channel only when it is in the open state [124–127]. Compared with the competitive inhibitors, which act on the glutamate or glycine site, and act more like an off switch, the uncompetitive channel blockers act more like a gain control [128]. Thus, they are proposed to work better for blocking pathologically excessive NMDAR activity without affecting its basal physiological activity, when their affinity and trapping time fall within the right range [128,129].

In comparison to other inhibitors in the channel blocker group, ketamine has a relatively moderate affinity, weaker than MK-801 and PCP, but stronger than memantine [130,131]. Thus, the superior efficacy of ketamine may be rooted in its unique mode of inhibition and optimal pharmaceutical properties. As we will see below, the antidepressant efficacy of many other NMDAR inhibitors is comparable to ketamine in preclinical studies (table 1). In clinical studies, their efficacy is suboptimal, except AXS-05 (table 2).

### (a) Preclinical data

In preclinical studies, many NMDAR inhibitors exert rapid antidepressant effects within 15 min to 1 h. These inhibitors include the competitive antagonists at the glutamate site, such as AP-7 [30], CGP37849 [32], CPPene (midafotel) [15]; competitive antagonists at the glycine site, such as ACPC [30], AV-101 [47] and L-701324 [72]; antagonists at the polyamine site, such as eliprodil [31]; GluN2B-specific allosteric antagonists, including Ro-256981 [13], CP-101,606 [73] and ifenprodil [74]; as well as open-channel blockers at NMDAR PCP/Mg^2+^ site, including ketamine [13,15,17,48], methoxetamine (MXE) and its analogues [49,50], MK-801 [15,51,52,61], lanicemine (AZD6765) [53], dextromethorphan (DM) [54,69–71] and memantine [64–66,68]. Moreover, some of these drugs, e.g. CPPene (midafotel) [15], AV-101 [47], L-701324 [72], Ro-256981 [58], ifenprodil [74], MK-801 [62,63], methoxetamine [49], D-methadone [56] and ketamine [13–15,57] have also been demonstrated to show antidepressant effects in a sustained fashion at 24 h or even later (table 1). Interestingly, MK-801 did not affect the performance in the FST test 24 h after injection in naive animals [13,15,55] but did show lasting antidepressant effects in both the FST and sucrose preference test (SPT) in depression-like mice [62,63], suggesting that the state of animals may be important to reveal the drug effect. This is an important point that we will reiterate in §6.

### (b) Clinical data

#### (i) Ketamine-type *N*-methyl-d-aspartate receptor channel blockers

Among the uncompetitive NMDAR antagonists that share the same blocking nature with ketamine, MK-801 and PCP are not clinically practicable, because they block normal physiological functions of NMDARs owing to super strong affinity and long ‘dwell time’ in the ion channel pore (slow off-rate) [128]. They can induce coma, hallucination and severe risks of abuse [128], which limit their clinical use.

Memantine, as a low-affinity channel blocker of NMDARs, has a faster off rate and lower trapping rate compared to ketamine [128,129,132]. This property lowers the potential for side effects but may also reduce its efficacy [57]. In addition, clinically memantine is always delivered orally, a route with a much larger first-pass effect (drug loss during absorption by liver and gut wall) that results in much lower plasma concentration (18% of intravenous ketamine under the same dose; [133–135]). Indeed, in line with the idea of emphasizing the importance of the delivery route, oral administration of ketamine capsules showed a substantially inferior response than intravenously injected ketamine, with minimal benefits even after two to six weeks of treatment [136]. These factors may account for the controversial antidepressant efficacy of memantine reported clinically (table 2; [83,86,87,90,137]). Under a relatively low dose (starting at 5 mg d^−1^ and titrated for 3 weeks up to 20 mg d^−1^), one double-blind randomized controlled trial (RCT) in MDD patients observed antidepressant efficacy of memantine comparable to the SSRI escitalopram [83]. However, using the same dosing regimen, three other double-blind RCTs in MDD patients did not show superior efficacy of memantine compared to placebo [84,85,137], even though one of these two trials did observe antidepressant effects during the first four weeks when the dose was being titrated up and also observed surprisingly high remission rate in the sixth week (memantine 64% versus placebo 27%; [85]). On the other hand, clinical results with higher doses of memantine showed more promise. A recent double-blind RCT with a higher initial dose (10 mg d^−1^) and a faster titration protocol (titrated for only 1 week up to 20 mg d^−1^) showed significant antidepressant effects of memantine compared to placebo in two weeks [87]. More clinical studies are needed in the future to clarify the effects of memantine. Ideally, the dosage and delivery route (e.g. intravenous versus oral) need to be optimized.

REL-1017 (esmethadone) is the inactive enantiomer of methadone and functions as an NMDAR pore blocker, like ketamine. In a recent double-blinded phase II clinical trial, it demonstrated a rapid (day 4) and sustained (day 14) antidepressant effect [94].

Another low-affinity NMDAR channel blocker, lanicemine, was first revealed in a double-blind RCT to show rapid but transient antidepressant activity within 80–110 min post a single infusion [91]. Other double-blind RCTs discovered a much longer antidepressant action of lanicemine throughout 10–13 days post a single infusion [92]. Such efficacy may be maintained for five weeks with multiple intermittent drug infusions [92]. However, a subsequent larger-scale clinical trial did not show superiority compared to the placebo, although both placebo and lanicemine groups of patients exhibited significant improvements [93].

DM, as a common antitussive and a Food and Drug Administration-approved drug for pseudobulbar affect [138], is also a potent NMDAR open-channel blocker, with a rapid off-rate similar to memantine [130,139]. Oral delivery of DM is convenient and safe [138,140]. According to the NMDAR hypothesis, it was proposed that DM may produce a rapid antidepressant response [131,141]. A previous open-label pilot study showed that DM (combined with quinidine to increase the bioavailability of DM) has antidepressant efficacy but is far from rapid, with a four-week onset delay [88,89]. Encouragingly, after increasing the daily dosage of DM (from 20 to 45 mg d^−1^) and adding bupropion which slows down the metabolism of DM, a larger-scale phase II double-blind RCT unravelled that as early as in one week, AXS-05 (formulated combination of DM and bupropion) exhibits superior antidepressant effects compared to control [22,23,88]. This promising result was further confirmed in a recent phase III double-blind RCT [21]. Such results again highlight the importance of dosage when evaluating novel antidepressants based on the NMDAR hypothesis and suggest the feasibility to strengthen the effectiveness of ketamine-like antidepressants by increasing their bioavailability.

#### (ii) Non-ketamine-type *N*-methyl-d-aspartate receptor inhibitors

Among non-channel-blocker-type NMDAR inhibitors, the GluN2B selective allosteric inhibitors, CP-101,606 (traxoprodil), MK-0657 show significant antidepressant efficacy in pilot clinical trials (table 2; [95,96,142]). The onset of their actions is from 24 h to 5 days [95,96,142]. However, a later trial (ClinicalTrials.gov Identifier: NCT01941043) using higher doses (20 mg d^−1^) of MK-0657 failed to show the expected efficacy, and current clinical trials of CP-101,606 have been discontinued owing to associated cardiovascular toxicity [36].

Inhibitors at the NMDAR glycine site also inspired extensive clinical studies [20], which have mostly focused on DCS and AV-101 [98]. DCS antagonizes NMDARs as competitive partial glycine site agonists [20,29] and was formulated as NRX- 101 (combined with lurasidone) by NeuroRx. AV-101 (L-4-chlorokynurenine) is a prodrug for 7-Cl-kynurenic acid, which functions as an antagonist at the NMDAR glycine site [143]. The clinical trials on these two antagonists are mixed with most trials showing negative results [27,28,97,98].

Nitrous oxide (N_2_O) is also an NMDAR antagonist, with an unknown binding site. In TRD patients, nitrous oxide displays promising efficacy, rapidly attenuating depression within 2 h and lasting for 24 h [99]. This was further confirmed by a follow-up phase II clinical trial [100].

In summary, there is a large body of evidence supporting the antidepressant activity of various NMDAR inhibitors in preclinical animal studies. Clinically, although other NMDAR antagonists (except AXS-05) have not yet proven as efficacious as ketamine, detailed analysis of individual cases reveals that some of these NMDAR inhibitors, especially ketamine-like channel blockers, have the promise to show antidepressant efficacy. It is worth noting that negative clinical results may be complicated by factors such as the placebo effect [144], pharmacological properties and therapeutic windows of each drug. Among the clinically significant antagonists, although most of them do not have a rapid effect comparable to ketamine (in a day), their antidepressant onset (in a week) is still significantly faster than traditional antidepressants. Currently, multiple NMDAR inhibitors are undergoing clinical investigations including REL-1017 (ClinicalTrials.gov Identifier: NCT06011577), NRX-101 (ClinicalTrials.gov Identifier: NCT03395392; NCT03396068) and nitrous oxide (ClinicalTrials.gov Identifier: NCT03869736; NCT05357040; NCT05710887; NCT05007028; NCT03167905).

## 4. Current arguments against *N*-methyl-d-aspartate receptor-based mechanisms of ketamine

In light of ketamine’s rapid and strong antidepressant effects, understanding its underlying mechanisms is of crucial importance and should help design better and safer treatments for depression. Debating on the major molecular target that mediates ketamine’s antidepressant effects, the field is currently divided into two camps: the NMDAR-dependent and the NMDAR-independent camps. For the ‘NMDAR-independent camp’, three major arguments have been raised to dispute the NMDAR-based mechanism of ketamine. Below we review these arguments and related evidence.

### (a) Failure of clinical trials of other *N*-methyl-d-aspartate receptor inhibitors

The first argument is that most alternative NMDAR inhibitors do not show similar antidepressant effects as ketamine [20]. However, as reviewed in §3, we can appreciate that: (i) many NMDAR inhibitors do show rapid and sustained antidepressant efficacy in animal studies; (ii) in clinical studies, the non-unanimous clinical efficacy of NMDAR inhibitors may be accounted for by their different inhibitory mechanisms, pharmacological properties, therapeutic windows, dosing regimens or routes of administration [129,132,145,146]; and (iii) although not as efficacious or as consistent as ketamine, some of these inhibitors (e.g. CP-101,606, memantine, lanicemine, REL-1017 and N_2_O) also caused antidepressant responses in some clinical tests. In particular, the recent success of the clinical phase III trial of a pore-blocking type NMDAR inhibitor, AXS-05 [21], has reinforced the notion that the pore domain of NMDAR is a promising target for antidepressant treatment.

### (b) (R)-ketamine-based argument

While ketamine was initially administered clinically as a (R, S) racemic mixture, its enantiomer (R)-ketamine, has lower affinity (by 2–4-fold) for NMDARs than the other enantiomer (S)-ketamine *in vitro* [12,102,147,148], yet was reported to exhibit more potent antidepressant effects than (S)-ketamine in animal studies [55,59,60]. This dissociation in affinity and potency was used as another argument to refute NMDAR-dependent mechanisms of ketamine’s antidepressant actions [55,60]. However, such an argument may be misleading, because for *in vivo* efficacy of any drug, affinity is only one of many relevant factors. For example, a drug with a lower affinity but a slower off-rate binding to the receptor can be more potent than a high-affinity drug that comes off faster from the target site, or a lower-affinity drug that absorbs better, eliminates slower or crosses the blood–brain barrier more efficiently can also show higher efficacy than a higher-affinity one. As of now, a full comparison between the two ketamine enantiomers has not been made for their pharmacological properties, including pharmacokinetics (absorption, distribution, metabolism and elimination) and pharmacodynamics (e.g. ligand-receptor binding properties, kon and koff). These parameters can all contribute to a drug’s potency and have not been taken into account by the current (R)-ketamine-based argument.

In addition, the argument was made based on data from animal studies. Further clinical trials are needed to rigorously compare the antidepressant efficacy among (R)-ketamine, (S)-ketamine and racemic ketamine. Current clinical data on (S)-ketamine showed comparable or even better efficacy to racemic ketamine in TRD patients [44,75–80,149,150], suggesting that clinically (S)-ketamine is not necessarily less potent than (R)-ketamine. On the other hand, clinical trials on (R)-ketamine show mixed results [81,82], with the most recent one showing no significant difference compared to placebo [82].

### (c) Ketamine-metabolite-based argument

Ketamine is metabolized in the liver into a series of metabolites. In a recent influential study, one such ketamine metabolite, (2R,6R)-HNK (referred to as HNK hereafter), was reported to evoke rapid and sustained antidepressant effects in mouse models of depression [55]. These effects were repeated later in some [48,119,151–153] but not other [154,155] studies, although the gender of experimenters was suggested to have an impact on the results [156]. At the concentration used in these studies (10 mg kg^−1^, intraperitoneal (i.p.)), it was argued that HNK does not block NMDARs (however, see [157] and below). Importantly, using a deuterated, metabolically inert form of ketamine, which has a much lower (30%) metabolism into HNK but still blocks NMDARs, Gould *et al*. observed in mice diminished sustained antidepressant effects, although the rapid antidepressant effects were still intact [55]. Based on these data, it was argued that the sustained antidepressant effects of ketamine may not depend on the blockade of NMDARs, but on its metabolite HNK [24,158], even though the rapid antidepressant effects cannot be accounted for by the latter.

Since HNK lacks some side effects (e.g. dissociation and addictive risk) associated with ketamine in animal models, it represents a promising new drug for exploration at the clinics. However, one debate on HNK centres around whether it blocks NMDARs or not at the treatment-effective concentration. In brain slices *in vitro*, HNK can block NMDARs at 50 μM [157] but not at 10 μM [55,157]. Liquid chromatography-tandem mass spectrometry of *in vivo* HNK level after i.p. injection (10 mg kg^−1^) indicates a peak concentration of 20 μM [159]. This concentration has not yet been tested for NMDAR blockade. Another debate on HNK regards the drug concentration. Only about 1/10 ketamine can be metabolized into HNK *in vivo* [55,102]. Therefore, the treatment-relevant dosage of ketamine (10 mg kg^−1^, i.p [17,160] can only be converted to about 1 mg kg^−1^ HNK in mice. This is much lower than the minimal dosage of HNK (10 mg kg^−1^, i.p.) required to produce antidepressant effects [55]. Such discrepancy argues that even though HNK itself may have antidepressant activity, it is unlikely that HNK mediates ketamine’s effects.

Moreover, similar to (R)-ketamine, current data on HNK’s behavioural effects are solely derived from animal studies. The clinical efficacy of HNK awaits future evaluation [129].

## 5. Ketamine’s other molecular targets in its antidepressant action—preclinical and clinical evidence

While recognizing ketamine as a high-affinity, high-potency inhibitor of NMDARs [103,104], we should also bear in mind that ketamine has multiple other molecular targets [102], including opiate receptors (Ki: 4.38–272 μM) [109–112], dopamine D2 receptors (D2Rs) (EC50: 0.4–2 μM; Ki: 0.05–1 μM) [106,117,118], tyrosine kinase receptor 2 (TRKB) (Ki: 2.86–12.3 μM) [119], serotonin 5-HT2 receptor (Ki: 15 μM) [106], serotonin transporter (IC50: 18.8 μM) [107], hyperpolarization-activated cyclic nucleotide-gated channel (HCN) channel (IC50: 8.2–15.6 μM) [108], cholinergic receptors (IC50: 17–92 μM for most nAChR subtypes; IC50: 5.7 μM for M1 mAChR; IC50: 3–9.5 μM for α3β4 nAChR) [105,113,114], oestrogen receptors (ERs) (EC50: 2.3 μM) [115] and dopamine transporters (DATs) (IC50: 4.6 μM) [116]. Compared with NMDARs (IC50: 0.4–8.2 μM; Ki: 0.18–4.9μM) [102–104], most of these other targets have a much lower affinity or inhibitory potency for ketamine, except for D2Rs and TRKB, which have comparable binding affinity to NMDARs.

Could activation of D2Rs or TRKB account for ketamine’s antidepressant effects? There is evidence that D2R agonists show rapid antidepressant activity in the FST (0.5–1 h post subcutaneous (s.c.) administration) [161], and that activation of D2Rs but not D1Rs is necessary for the rapid antidepressant actions of ketamine in the FST in mice [162]. Clinically, D2R agonists exert slow antidepressant actions after several weeks of daily administration [163–165]. However, departing from early assessments [106,117,118], a recent study, using a G protein-coupled receptor (GPCR)-based functional binding assay, revealed that ketamine does not interact with D2Rs under treatment-relevant dosage [166]. Therefore, it remains to be determined whether D2Rs play a direct role in mediating ketamine’s rapid antidepressant effects.

TRKB was recently reported to be a binding partner of SSRIs, ketamine and psychedelics [119,167]. However, one key question remains to be addressed from these studies. That is, given that SSRIs have an even higher binding affinity to TRKB [119] and a higher or similar brain concentration after a single drug administration [102,168–170] compared with ketamine, why don’t SSRIs show antidepressant effects as rapidly as ketamine?

Among the lower-affinity targets, the opioid receptors are the most studied, especially the μ opioid receptors, which have 10–100-fold lower affinity for ketamine than NMDARs [104,110]. As a partial agonist, the activation of μ opioid receptors was recently implicated in the sustained antidepressant effects of ketamine: in one pilot clinical study, pretreatment of the opioidergic antagonist, naltrexone, abolished the antidepressant effects of ketamine at 1–14 days post-infusion [171]. However, a follow-up clinical study [172] and another preclinical study [173] showed that both the rapid and sustained antidepressant effects of ketamine were not attenuated by naltrexone pretreatment. On the other hand, compared with opioid agonists, ketamine’s affinity for the opioid receptors is 10 000-fold lower [110]. If acute activation of opioid receptors was required for ketamine’s antidepressant actions, the concomitant use of high-affinity opioid agonists should occupy the binding sites of opioid receptors and attenuate the efficacy of ketamine. Based on this rationale, a clinical study was conducted in patients receiving stable chronic opioid agonists and found that ketamine still drastically reduced depression scores [174].

Furthermore, in a rat model of depression, Malinow and colleagues found that opioid antagonists abolish the ability of ketamine to reduce lateral habenula (LHb) hyperactivity and depression-like behaviours; but activation of opiate receptors themselves does not produce antidepressant effects [175]. These results reiterated the idea that the opioid system does not mediate the actions of ketamine but rather plays a permissive role [175].

## 6. Concluding remarks and future perspectives

In conclusion, with the current evidence base, we believe that the NMDAR hypothesis remains a strong and appealing hypothesis to explain ketamine’s antidepressant actions. On the one hand, extensive preclinical data have demonstrated the antidepressant activity of various NMDAR inhibitors, some of which also show promise in clinical studies. Several mechanistically plausible models have been put forward to explain how blockade of NMDARs by ketamine may lead to antidepressant effects [15–17,132,176–190]. For example, an attractive model proposes that ketamine exerts its antidepressant effects by blocking NMDAR-dependent burst firing in the brain’s anti-reward centre, the LHb [17,57,176].

On the other hand, most current caveats and controversies regarding the NMDAR hypothesis of ketamine are based either on clinical data of alternative NMDAR inhibitors as antidepressants or on preclinical data of ketamine’s enantiomers or metabolites. Such arguments are thought-provoking, but for reasons discussed above, may be premature at the current stage. As evident in the case of alternative NMDAR inhibitors, there is often a gap in drug efficacy between preclinical and clinical studies, partly owing to pharmacokinetic and pharmacodynamic differences between rodents and humans. For this reason, any positive clinical data from drugs that share the same molecular or cellular targets of ketamine would be much more compelling in revealing ketamine’s mechanisms than arguments based on negative results. Future endeavours are needed to identify the key unique pharmacological feature(s) (e.g. binding affinity, channel trapping rate, noncompetitive and open-channel nature of blockade) of ketamine that enable its superb antidepressant activity. In addition, we would like to highlight the following points for future work addressing ketamine’s antidepressant mechanisms.

First, the brain region specificity of ketamine action warrants special attention. NMDARs are globally expressed in the brain, mediating different physiological functions depending on their locations, and being opened by different input stimuli depending on the behavioural states. Importantly, NMDARs in different brain regions may not be all inhibited by ketamine owing to its use-dependent blocking manner. At the basal state and at the time of ketamine infusion in depressed patients, as deduced from the data of animal [188,191,192] and human imaging studies [193], most brain regions are perhaps inactive and contain closed NMDARs. As an open-channel blocker, ketamine has much better accessibility to brain regions (e.g. LHb) or cell types (e.g. certain classes of interneurons) that are active and have NMDARs in the open state. Future studies on the molecular substrates of ketamine should be placed in the context of such neural circuits or cell types that are directly relevant to ketamine’s action.

Second, for the same reason above, it will be more relevant to use animals in the depression-like state instead of the naive state to evaluate the antidepressant effects of NMDAR inhibitors, especially the use-dependent type inhibitors (e.g. memantine) [67]. Under the naive state, depression-related brain circuits such as the LHb may be silent and not readily accessible to use-dependent inhibition. Another drawback of studies using naive animals is that such studies are often not comprehensive, usually only measuring despair-like phenotype using FST or the tail suspension test, but not anhedonia phenotype using SPT (owing to the ceiling effect). It will be useful to re-evaluate their effects in fuller spectrums on depression-like animals.

Third, given that different molecular or cellular substrates, or different brain regions may be recruited at different time points after ketamine infusion, it may be helpful to dissociate the rapid (hours) versus the sustained (days) antidepressant effects of ketamine and examine their underlying mechanisms accordingly. For example, whereas ketamine’s rapid effects may be mediated by the acute blockade of NMDARs, its sustained effects may either result from plasticity changes consequent on the acute blockade [145,182] or from long-term blockade of NMDARs [57]. Regarding the first possibility, in the context of the theme of long-term potentiation (LTP) in the current issue, it is worth noting that three independent studies published in 1983 showed that ketamine is an NMDAR antagonist [8], that ketamine blocks the induction of LTP [194] and that NMDARs mediate the induction of LTP [195]. It will be relevant to examine whether inhibition of NMDARs impacting synaptic plasticity in reward circuits may contribute to ketamine’s persistent antidepressant action. Towards the latter possibility, although ketamine has a short plasma half-life, it also has a slow off rate. Current data on ketamine’s trapping rate from the NMDARs is mostly derived from dissociated neuronal cultures [102,196,197]. Determining how long the trapped ketamine can continue to block the NMDARs in the relevant brain circuits *in vivo* is of critical importance and will provide valuable information for understanding ketamine’s sustained antidepressant effects [57]. Recently, we discovered in mice that a single administration of ketamine could effectively block the NMDAR in the LHb for up to 24 h, a duration significantly longer than its elimination half-life (approximately 13 min) [57]. This trapping effect may play an important role in the sustained antidepressant effects of ketamine and holds strong therapeutic implications.

Last but not least, the potential synergistic effect of NMDARs with ketamine’s other molecular targets needs to be further considered. As discussed above, among the host of ketamine’s molecular targets, some of them have the IC50 or EC50 in the range of 0.4–3 μM [105,115,117]. With i.v. infusion in human patients, the peak plasma concentration of ketamine can reach as high as 1–1.5 μM [102,133,198], which is sufficient to at least partially inhibit or activate these other molecular targets. Currently, we cannot exclude the possibility that some of these alternative molecular targets may also be engaged, synergistically with NMDARs, to contribute to ketamine’s antidepressant effects. Although speculative at this point, this may be another plausible reason why ketamine has more superior antidepressant effects than other NMDAR inhibitors. Investigation into such possibilities may open new avenues for novel treatments of depression.

## Data Availability

This article has no additional data.
